# Genome-wide investigation of *in vivo *EGR-1 binding sites in monocytic differentiation

**DOI:** 10.1186/gb-2009-10-4-r41

**Published:** 2009-04-19

**Authors:** Atsutaka Kubosaki, Yasuhiro Tomaru, Michihira Tagami, Erik Arner, Hisashi Miura, Takahiro Suzuki, Masanori Suzuki, Harukazu Suzuki, Yoshihide Hayashizaki

**Affiliations:** 1 RIKEN Omics Science Center, RIKEN Yokohama Institute 1-7-22 Suehiro-cho, Tsurumi-ku, Yokohama, Kanagawa 230-0045, Japan; 2 International Graduate School of Arts and Sciences, Yokohama City University, 1-7-29 Suehiro-cho, Tsurumi-ku, Yokohama 230-0045, Japan

## Abstract

**Background:**

Immediate early genes are considered to play important roles in dynamic gene regulatory networks following exposure to appropriate stimuli. One of the immediate early genes, *early growth response gene 1 *(*EGR-1*), has been implicated in differentiation of human monoblastoma cells along the monocytic commitment following treatment with phorbol ester. EGR-1 has been thought to work as a modifier of monopoiesis, but the precise function of EGR-1 in monocytic differentiation has not been fully elucidated.

**Results:**

We performed the first genome-wide analysis of EGR-1 binding sites by chromatin immunoprecipitation with promoter array (ChIP-chip) and identified EGR-1 target sites in differentiating THP-1 cells. By combining the results with previously reported FANTOM4 data, we found that EGR-1 binding sites highly co-localized with CpG islands, acetylated histone H3 lysine 9 binding sites, and CAGE tag clusters. Gene Ontology (GO) analysis revealed enriched terms, including binding of molecules, in EGR-1 target genes. In addition, comparison with gene expression profiling data showed that EGR-1 binding influenced gene expression. Moreover, observation of *in vivo *occupancy changes of DNA binding proteins following PMA stimulation indicated that SP1 binding occupancies were dramatically changed near EGR-1 binding sites.

**Conclusions:**

We conclude that EGR-1 mainly recognizes GC-rich consensus sequences in promoters of active genes. GO analysis and gene expression profiling data confirm that EGR-1 is involved in initiation of information transmission in cell events. The observations of *in vivo *occupancy changes of EGR-1 and SP1 suggest that several types of interplay between EGR-1 and other proteins result in multiple responses to EGR-1 downstream genes.

## Abstract 2

A Genome-wide analysis of EGR-1 binding sites reveals co-localization with CpG islands and histone H3 lysine 9 binding. SP-1 binding occupancies near EGR-1 binding sites are dramatically altered.

## Background

Regulatory gene networks, involving specific DNA elements and various transcription regulators, control living cells. To maintain a stable cellular state, multiple cell type-specific transcription regulators interact with DNA binding sites in target genes. For example, enforced expression of four transcription factors (MYC, OCT3/4, KLF4 and SOX2) in differentiated cells drives pluripotent-specific gene expression and is capable of maintaining pluripotency and self-renewing characteristics [1]. On the other hand, the molecular mechanism for cell state changes following exposure to appropriate stimuli has not been fully elucidated, although the induction of a set of immediate early genes is thought to constitute the first step in the cellular molecular response to stimulant signals for state changes.

Early growth response gene 1 (EGR-1; also known as NGFI-A, KROX-24, ZIF268 or TIS8) contains a highly conserved DNA-binding domain composed of three C_2_H_2 _classical zinc finger motifs that belongs to the immediate early gene family. EGR-1 is rapidly and transiently induced by various stimulants, such as growth factors [2], neurotransmitters [3], hormones [4], stress [5] and injury [6], and recognizes a 9 base pair segment in GC rich regions in the promoters of target genes. EGR-1 is also involved in cell growth [7], synaptic activation [8], apoptosis in vascular cells [9] and mitogenesis [10]. Moreover, EGR-1 may play an essential role in cell differentiation along the monocyte lineage. Liebermann and colleagues [11] reported that antisense oligomers for Egr-1 blocked macrophage differentiation in myeloid leukemia cell lines and normal myeloblasts, and ectopic expression of Egr-1 in cell lines and primary bone marrow resulted in activation of the macrophage differentiation program [12,13]. However, the precise function of EGR-1 in monocyte differentiation has not been clearly defined.

Recently, we analyzed the transcriptional network in differentiation of human myelomonocytic leukemia THP-1 cells as a system model following treatment of phorbol 12-myristate 13-acetate (PMA) using data from the FANTOM4 consortium [14]. Our analysis using FANTOM4 data, including microarrays of mRNA, deepCAGE and chromatin immunoprecipitation with genome tiling array (ChIP-chip) [15], revealed that cellular states were constrained by complex networks involving substantial numbers of both positive and negative regulators. In this study, in order to investigate EGR-1 function during monocyte differentiation, genome-wide EGR-1 binding site data were produced using ChIP-chip and integrated with the available FANTOM4 data. Consequently, we present a whole-genome EGR-1 binding profile and propose possible functions of EGR-1.

## Results

### EGR-1 expression during THP-1 differentiation

**Figure 1:**
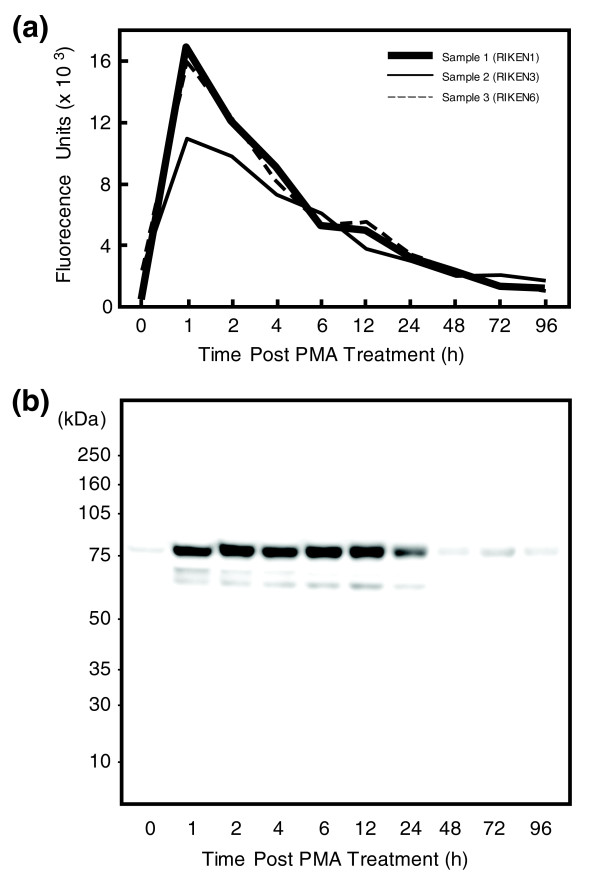
EGR-1 expression during THP-1 differentiation. **(a) **Quantile normalized *EGR-1 *transcript levels were produced by Illumina Human Sentrix-6 bead chips v.2. **(b) **EGR-1 protein levels by western blotting using an EGR-1 polyclonal antibody.

To assess whether the expression of EGR-1 in THP-1 cells changes during the time course of monocyte differentiation following PMA stimulation, we analyzed microarray data in the FANTOM4 data sets (see Materials and methods). *EGR-1 *mRNA was up-regulated immediately after PMA treatment, reaching a maximum at 1 hour and decreasing dramatically thereafter (Figure 1a). Also, quantitative RT-PCR analysis indicated that *EGR-1 *mRNA in THP-1 cells was transiently induced by PMA stimulation (data not shown). These observations of mRNA changes were similar to those reported previously using HL60 and primary human monocytes [16]. Moreover, western blotting using an EGR-1 polyclonal antibody assessed levels of EGR-1 protein in nuclear extracts from untreated and PMA-stimulated cells (Figure 1b). As expected, small amounts of EGR-1 protein were detectable in the untreated state, while EGR-1 translation at 1 hour after stimulation was drastically elevated and returned to pre-stimulation levels by 48 hours. The EGR family members, including EGR-1, EGR-2, EGR-3, EGR-4 and WT-1, share a highly homologous DNA binding domain and three or four zinc finger motifs. However, since the flanking regions of the EGR family are much less conserved and the molecular sizes of all EGR proteins but EGR-1 are less than 55 kDa, the polyclonal antibody against EGR-1 was judged to cross-react with negligible amounts of other EGR family proteins. These results show that *EGR-1 *mRNA and protein were significantly and transiently expressed soon after PMA stimulation.

**Figure 2:**
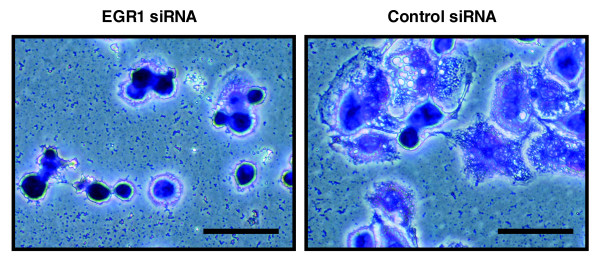
Effect of siRNA against *EGR-1 *in THP-1 differentiation. Photographs show typical morphological changes by Giemsa stain in *EGR-1 *or control siRNA transfected THP-1 cells at 48 hours after PMA stimulation. Scale bar = 50 μm.

To test the essential role of EGR-1 in THP-1 differentiation reported previously [11], RNA interference was employed to specifically knockdown the EGR-1 mRNA. The small interfering RNA (siRNA) for EGR-1 was designed against a target sequence located at the 3' end of the EGR-1 coding region and conjugated with Alexa Fluor 555. Quantitative RT-PCR was then used to verify siRNA-mediated down-regulation of *EGR-1 *mRNA (Additional data file 1a). THP-1 cells were treated with either *EGR-1 *siRNA or a negative control siRNA and exhibited a similar efficiency of transfection (Additional data file 1b, upper). Fourty-eight hours after transfection prior to PMA stimulation, there was no detectable difference in morphology between *EGR-1 *siRNA-treated cells and the negative control. Moreover, a couple of hours after PMA treatment, both the treated and control cells adhered to the culture dish. However, inhibition of THP-1 differentiation by *EGR-1 *knockdown was observed at 48 hours after PMA stimulation (Figure 2 and Additional data file 1b, lower). Taken together, these data indicate that EGR-1 has an important role during monocyte differentiation in THP-1 cells as well as other myeloid leukemia cell lines and normal myeloblasts.

### Identification of EGR-1 binding sites in CpG islands

**Figure 3:**
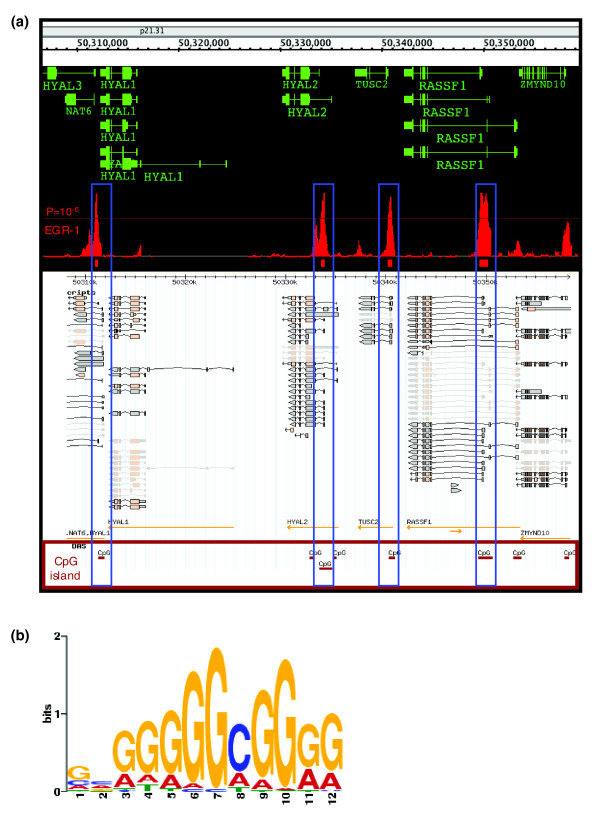
Co-localization of EGR-1 binding sites with CpG islands. **(a) **RefSeq genes, and ChIP-chip data of EGR-1 and CpG island location are shown (positions 50,306,500 to 50,359,500 of human chromosome 3). Signal-enriched regions on CpG islands are highlighted in blue boxes. **(b) **The most overrepresented sequence identified by MEME analysis (*E*-value = 7.5e-087).

Although EGR-1 is thought to be a DNA binding protein with three zinc finger motifs, and reported target genes have been studied using single gene approaches such as reporter and gel shift assays, EGR-1 binding sites have previously not been studied on a whole genome basis. In order to identify novel target genes or DNA binding sites in the context of the genome around transcriptional start sites (TSSs), we performed ChIP-chip analysis as a comprehensive and unbiased approach. Since we hypothesized that EGR-1 would exert its direct effects on transcriptional regulation by binding promoter regions, human promoter arrays covering approximately 7.5 kb upstream through 2.45 kb downstream of 5' TSSs of approximately 25,500 genes were used. For hybridization, we prepared immunoprecipitated chromatin samples from THP-1 cells treated with PMA for 1 hour. Members of the immediate early genes family, including EGR-1, are believed to constitute the first step in transcriptional regulation and operate in a hierarchical manner by induction of expression of downstream factors. Therefore, we predicted that a small number of binding sites of EGR-1 would be detected in the array. Surprisingly, however, many were observed. For identification of high confidence EGR-1 binding sites on the human promoter arrays, we chose clusters where overlapping sites in biological replicates had over five consecutive array probes with a *P*-value < 1e-6 (see Materials and methods). Using these criteria, we identified 3,301 clusters, and noticed that these clusters overlapped the promoters of known EGR-1 target genes, such as those encoding TNF, NAB2, ID3 and SOD1 [17-20], as well as myeloid related genes (Additional data file 2). Based on previous reports [21] that EGR-1 recognizes a GC rich consensus sequence (5'-WTGCGTGGGCGK-3'), we predicted that EGR-1 binding sites would localize to CpG islands to a high extent. Thus, to assess whether EGR-1 and CpG islands co-localized, we compared putative EGR-1 binding loci with the locations of CpG islands obtained from the UCSC Genome Browser database (Figure 3a). The putative EGR-1 loci were localized to CpG islands in 77.8% of the cases.

**Figure 4:**
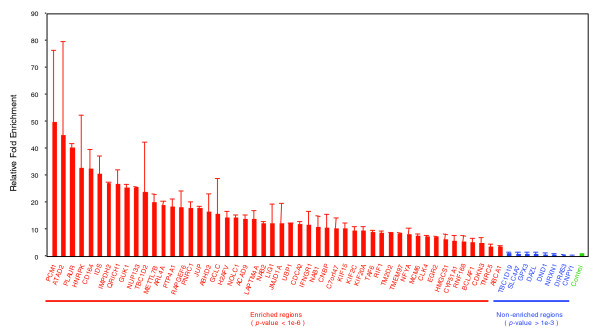
Validation of EGR-1 enrichment by ChIP-real-time PCR analysis. PCR primers were designed to 50 regions in selected clusters and 8 negative regions without enrichment in CpG islands. Data are relative fold enrichments, calculated by determining the apparent immunoprecipitation efficiency and normalized to the level observed at a control region (mean ± standard deviation, n = 2).

To search for significantly overrepresented DNA sequences in the putative EGR-1 binding loci, we used the multiple Em for motif elicitation (MEME) method. Due to input data size limitations of the web-based MEME application (version 4.1.0) [22], we randomly selected and analyzed 271 loci (87,782 bases) out of 3,301. The most highly overrepresented sequence provided by the MEME analysis (*E*-value = 7.5e-087) was similar to the previously reported EGR-1 motif (Figure 3b). In order to validate the criteria used above, we prepared new independent ChIP samples and performed ChIP-real-time PCR analysis against 50 regions in selected clusters and 8 negative regions without enrichment in CpG islands. We observed that all of the 50 regions showed higher enrichment (3.4- to 49.5-fold) than that in negative regions (0.01- to 0.98-fold) (Figure 4 and Additional data file 3). Thus, we used these criteria in the further analysis.

### Co-localization of EGR-1 with histone acetylation and transcription start sites

**Figure 5:**
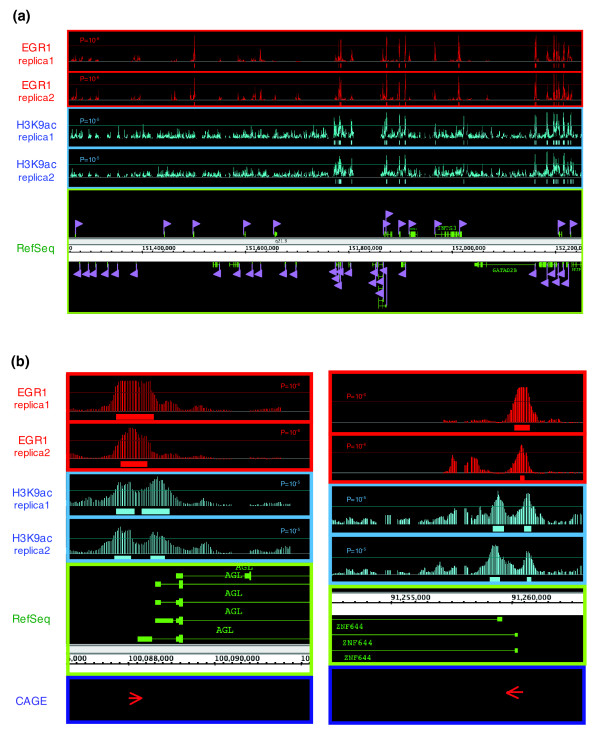
Identification of EGR-1 and H3K9ac enriched sites and CAGE tags in the human genome. **(a) **Examples of ChIP-chip data obtained with human promoter arrays (position 151,250,000 to 152,250,000 of human chromosome 1). Arrowheads indicate TSSs and direction. **(b) **EGR-1 co-localizes with H3K9ac and CAGE tags at the *AGL *and *ZNF644 *loci.

**Figure 6:**
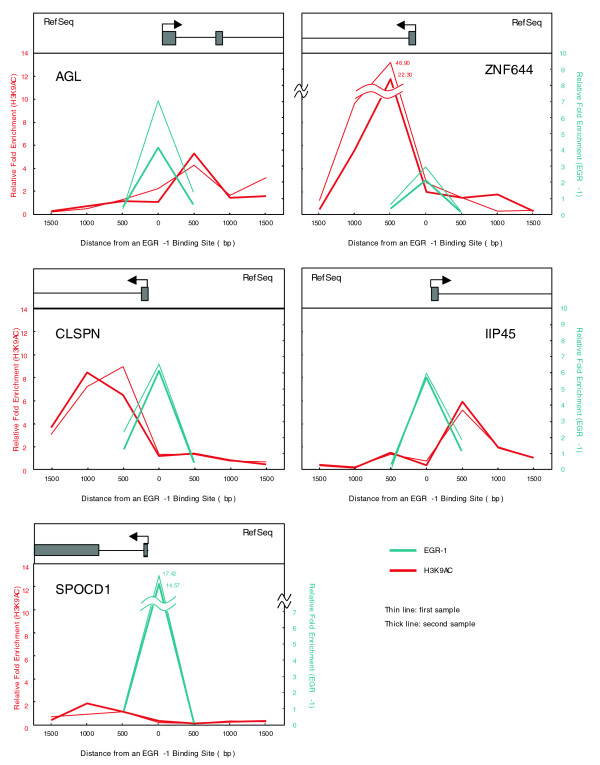
ChIP-real time PCR validation around EGR-1 enriched regions using THP-1 cell samples 1 hour after PMA treatment. Relative fold enrichment for H3K9ac (red) and EGR-1 (blue) are shown. Two independent experiments were performed, one represented by thin lines and one by thick lines. Gene start and direction of transcription are indicated by arrows.

Comparison of ChIP-chip data of EGR-1 with FANTOM4 data sets (see Materials and methods) revealed that EGR-1 co-localized with histone H3 lysine 9 acetylation (H3K9ac) sites in the chromatin samples that were prepared at 0 hour of PMA stimulation, prior to EGR-1 induction. As a typical case, direct comparison of EGR-1 and H3K9ac ChIP-chip data across a 1 Mb region of human chromosome 1 is shown in Figure 5a. The right side of the screenshot from the genome browser (human chromosome 1: 151,760,000 to 152,250,000 from build NCBIv36 [hg18]) shows that substantial enrichments for EGR-1 and H3K9ac are predominantly confined to sharp peaks and that many of these lie at the TSSs of annotated genes, while there is a low number of peaks to the left (chromosome 1: 151,250,000 to 151,760,000), even though several Refseq genes were annotated within this region. Since it is known that H3K9ac modification is tightly associated with the TSSs of genes, this observation indicated that EGR-1 binding would correlate with chromatin structure and/or gene expression. As more detailed examples, the nearest significant signals of EGR-1 and acetylation of H3K9 around the TSSs of AGL and ZNF644 are shown (Figure 5b). Two major peaks surrounding a TSS were detected for H3K9ac, and EGR-1 enrichment was observed around H3K9ac peaks, especially in the vicinity of TSSs. Interestingly, we also noticed that CAGE (cap analysis gene expression) tags co-localized with EGR-1 enrichments (Figure 5b). CAGE is a unique and original TSS identification method that samples 20- or 21-nucleotide sequence tags derived from the proximity of the cap site of mRNA [23]. Based on the potential EGR-1 binding regions derived from the above criteria, we examined the association of the 3,301 EGR-1 clusters with H3K9ac enriched loci and found that more than 75% of EGR-1 binding regions were located within 500 bp of H3K9ac enriched loci (Additional data file 4). Moreover, we observed that 69% of EGR-1 binding regions were located within 2 kb of CAGE tag clusters. Together, 87% of EGR-1 binding regions were associated with either H3K9ac or CAGE tag clusters. To verify the status of H3K9ac after PMA stimulation, ChIP-real-time PCR was carried out by using two EGR-1/H3K9ac enriched regions (AGL and ZNF644) and three EGR-1 enriched regions without H3K9ac enrichments (CLSPN, IIP45 and SPOCD1). As shown in Figure 6, high levels of H3K9ac around EGR-1 enrichments were observed, including two out of the three H3K9ac negative regions before PMA stimulation, thus demonstrating new enrichment of H3K9ac. In summary, EGR-1 binding was shown to be highly correlated with acetylation of H3K9 and TSSs of expressed genes, which suggests that gene activation is important for EGR-1 target site selection.

### Gene Ontology enrichment analysis of EGR-1 target genes

In order to further elucidate the functions of EGR-1 target genes, we examined gene ontologies using the web-based analysis tool GOstat [24,25]. For 3,301 EGR-1 clusters fully or partly overlapping RefSeq TSSs within ± 1 kbp, Entrez gene names were collected. We obtained 2,705 genes in this way, including several cases where the same cluster overlapped the TSS region of more than one gene. In the GOstat analysis, the 2,705 genes were compared to 17,142 genes as background that were identified by the same clustering method with a *P*-value of 1. Interestingly, the statistically significantly overrepresented Gene Ontology (GO) biological process terms were highly enriched for nucleic acid-related words such as gene expression and RNA processing (Table 1). Moreover, with regard to GO molecular function terms, the EGR-1 target genes list included binding of nucleic acids and proteins (Table 2). Information transmission such as transcriptional and translational cascades begin with binding of molecules, followed by signal amplification through a combination of molecular interactions, so we conclude that the results of the GOstat analysis support the notion that EGR-1 acts as an initiator of information transmission in cell events.

**Table 1:** Enrichment of Gene Ontology biological process terms in ChIP hits with EGR-1

GO term IDs	Enriched GO terms	*P*-value
GO:0043170	Macromolecule metabolic process	8.73E-031
GO:0044238	Primary metabolic process	7.20E-029
GO:0044237	Cellular metabolic process	8.44E-028
GO:0043283	Biopolymer metabolic process	4.34E-026
GO:0006139	Nucleobase, nucleoside, nucleotide and nucleic acid metabolic process	6.82E-026
GO:0006259	DNA metabolic process	7.99E-017
GO:0010467	Gene expression	2.98E-016
GO:0015031	Protein transport	3.70E-014
GO:0016070	RNA metabolic process	1.04E-013
GO:0016071	mRNA metabolic process	1.16E-013
GO:0045184	Establishment of protein localization	2.55E-012
GO:0033036	Macromolecule localization	2.80E-012
GO:0005654	Nucleoplasm	3.77E-012
GO:0006396	RNA processing	3.77E-012
GO:0006281	DNA repair	7.32E-012
GO:0006886	Intracellular protein transport	7.66E-012
GO:0008104	Protein localization	4.26E-011
GO:0006260	DNA replication	4.73E-011
GO:0016043	Cellular component organization and biogenesis	1.30E-010
GO:0006974	Response to DNA damage stimulus	1.40E-010
GO:0006397	mRNA processing	2.05E-009
GO:0046907	Intracellular transport	3.40E-009
GO:0008380	RNA splicing	7.02E-009
GO:0007049	Cell cycle	2.81E-007
GO:0022613	Ribonucleoprotein complex biogenesis and assembly	9.37E-007

**Table 2:** Enrichment of Gene Ontology molecular function terms in ChIP hits with EGR-1

GO terms IDs	Enriched GO terms	*P*-value
GO:0003676	Nucleic acid binding	3.08E-019
GO:0003723	RNA binding	6.44E-018
GO:0005515	Protein binding	5.65E-009
GO:0000166	Nucleotide binding	3.84E-007
GO:0003677	DNA binding	5.69E-007
GO:0051082	Unfolded protein binding	1.72E-006

### The influence of EGR-1 occupancy on gene expression dynamics

**Figure 7:**
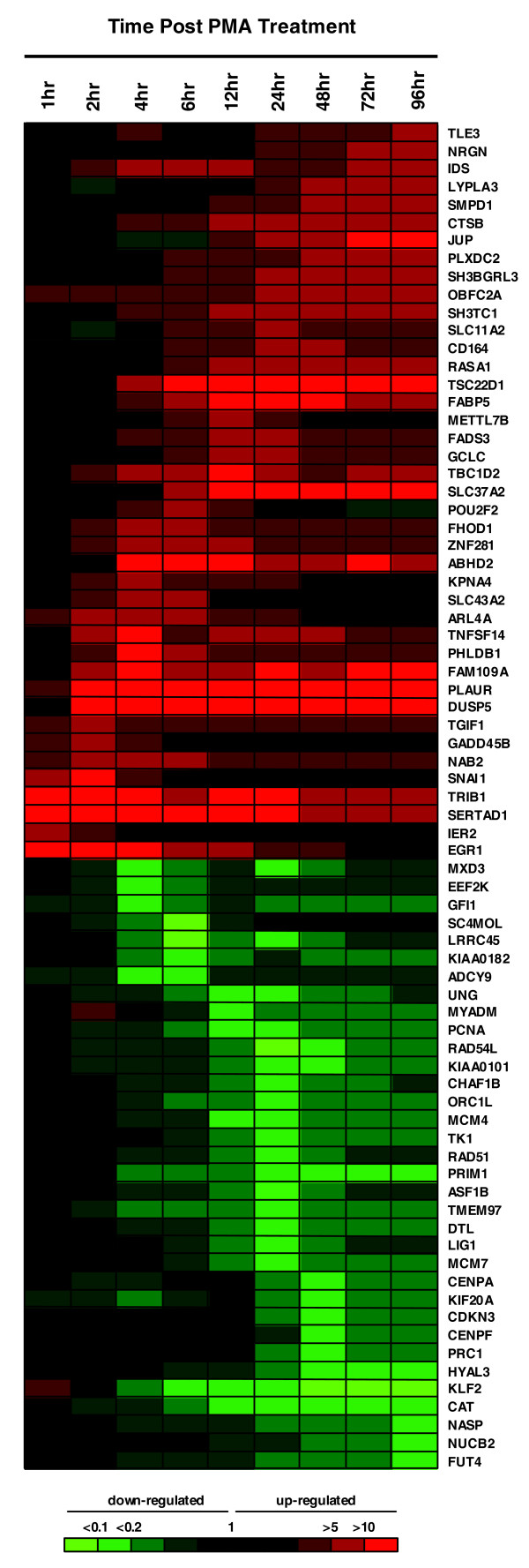
Expression profile of dynamically changed EGR-1 target genes over a period of 96 hours after PMA stimulation. Seventy-five genes, which changed expression relative to pre-stimulation by at least fivefold for at least one of the time points, are shown. Red, green and black denote increased, decreased and no change in gene expression.

To address whether EGR-1 binding at 1 hour after stimulation influenced expression of the target genes, mRNA microarray data in the FANTOM4 data sets, where the levels of various mRNAs were monitored over a time-course following PMA stimulation, were interrogated. In order to focus on genes with early dynamic expression changes, we identified genes that were up- or down-regulated at least five-fold at any time point within the first 6 hours after PMA stimulation, compared to the 0 hour initial time point. Out of 7,067 detectable genes during the whole time course, 209 were either up-regulated (145) or down-regulated (64) within 6 hours. Since 12 out of the 209 genes were not annotated in the human promoter array, 197 genes were then compared with the 2,705 EGR-1 target genes. Twenty-four up-regulated genes and eight down-regulated genes were found in the list of EGR-1 target genes and, as expected, immediately up-regulated genes were associated with EGR-1 binding in their promoter regions (Table 3). Five out of 21 (24%) and 7 out of 28 (25%) promoters of identified genes in the groups of up-regulated transcripts at 1 hour and at 2 hours, respectively, were observed to belong to EGR-1 target genes. In contrast, in the group of up-regulated transcripts after 4 hours and the group of down-regulated genes, we did not find similar enrichments of EGR-1 binding sites in immediately up-regulated genes (0-14%). The EGR-1 association with early up-regulated genes was not statistically significant (Fisher's exact test); however, the small *P*-value (*P *= 0.06) suggests that this may be due to the small sample size. Based on the western blot analysis (Figure 1b), we hypothesized that EGR-1 plays a role as an activator, and that the target gene expressions would be affected until 24 hours after EGR-1 induction, and return to basal levels thereafter. To verify this speculation, of the 2,705 EGR-1 target genes we identified 75 genes whose expression levels changed dynamically by at least five-fold for at least one time point over a time course between 0 and 96 hours after stimulation (Figure 7). Unexpectedly, the 75 genes contained not only transient up-regulated genes but also transient down-regulated genes and enhanced/suppressed genes at 96 hours after stimulation. These data suggested that EGR-1 binding affects multiple steps in the modulation of gene expression. We speculated, therefore, that multiple responses in gene expression by EGR-1 binding result from several types of interplay between EGR-1 and other proteins.

**Table 3:** Number of genes showing changes in early dynamic expression after PMA treatment with promoter regions that are bound by EGR-1

	Number of promoters bound at time points after PMA treatment			
	
	1 hour	2 hours	4 hours	6 hours
Up-regulated	5 (23.8%)	7 (25.0%)	9 (14.3%)	3 (12.5%)
Down-regulated	0 (0%)	0 (0%)	4 (13.8%)	4 (12.9%)

After PMA treatment, 136 genes were up-regulated and 61 down-regulated. Of these, 24 and 8, respectively, were found to be EGR-1 target genes. Percentage values indicate the number of target genes with bound promoters whose expression was up- or down-regulated at the indicated time point out of all EGR-1 target genes up- or down-regulated, respectively, over the entire time course (0-6 hours).

**Figure 8:**
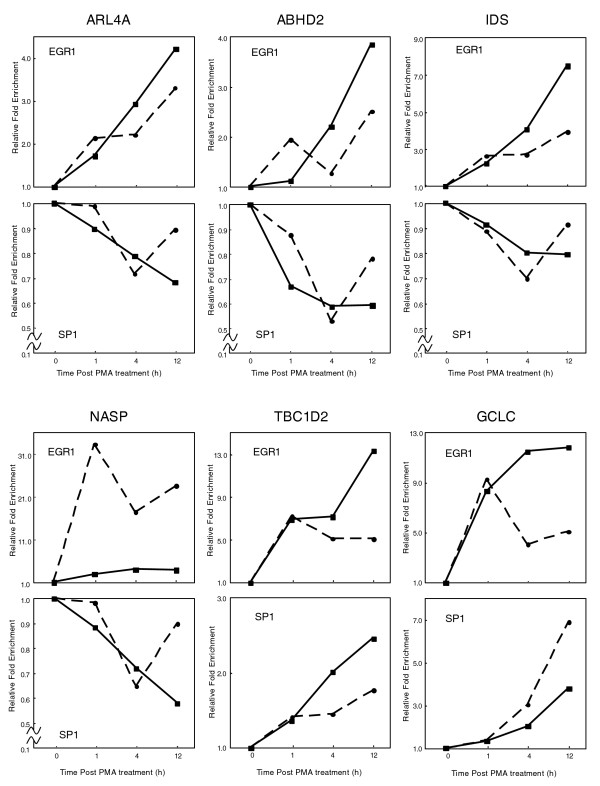
Relative occupancy changes of EGR-1 and SP1 in response to PMA stimulation. ChIP samples against EGR-1 or SP1 were prepared at the appropriate time, followed by real-time PCR of ChIP enriched DNA. Solid and broken lines show the relative fold enrichment of independent experiments.

To test the above speculation, the *in vivo *relationship between EGR-1 and SP1 in THP-1 differentiation was analyzed, since transcriptional regulation mediated through the interplay between EGR-1 and SP1 has been reported previously [26]. First, the protein level of SP1 was assessed by western blot analysis during PMA stimulation. Unlike EGR-1, we observed that SP1 expression gradually increased (Additional data file 5) throughout the time course. Second, to find SP1 sites coinciding with EGR-1 enriched loci, EGR-1 ChIP-chip data were compared to SP1 ChIP-chip results at PMA pre-stimulation, which had been produced previously as one of the FANTOM4 data sets (see Materials and methods). In this analysis, we found that 48-53% of EGR-1 sites were identical to SP1 sites with high confidence (Additional data file 6). In 75 dynamically changed EGR-1 target genes, we found that 34 loci (45.3%) were identical to SP1 sites. Finally, to examine the binding dynamics of EGR-1 and SP1 at the co-localized sites, six genes (*ARL4A*, *ABHD2*, *IDS*, *NASP*, *TBC1D2*, *GCLC*) out of the 34 identified loci were manually selected and the kinetics of EGR-1 and SP1 binding *in vivo *were assessed. By using ChIP-real-time PCR analysis, PMA treatment-induced EGR-1 binding at all examined loci was observed (Figure 8). ChIP experiments with anti-SP1 antibodies showed that SP1 binding occupancy in *TBC1D2 *and *GCLC *increased following PMA treatment, and indicated that SP1 occupancy in both loci was positively correlated with EGR-1 occupancy and the amounts of SP1 protein in the nucleus. On the other hand, SP1 binding occupancies in promoter regions of four genes (*ARL4A*, *ABHD2*, *IDS*, *NASP*) showed inverse relationships to EGR-1 occupancies.

## Discussion

Several transcription factors, especially EGR-1, have been implicated in differentiation of human monoblastoma cells along the monocytic commitment following treatment with PMA. EGR-1 has been thought to work as a modifier of monopoiesis, but it has not been clear where immediately induced EGR-1 is distributed throughout the genome. The results of the study presented here indicate that EGR-1 mainly recognizes GC-rich consensus sequences of active genes in CpG islands. CpG island promoters are most often associated with ubiquitously expressed genes, so-called housekeeping genes, but are also associated with many exceptions to this, including embryonic development and brain-specific genes [27,28]. Previous reports have shown that not only chromatin structure, but also DNA methylation in CpG islands, can control gene expression [29]. Ogishima *et al*. [30] reported that DNA hypomethylation within promoter CpG islands of the gene encoding heparanase facilitated EGR-1 binding to its consensus motif. Since DNA methylation in CpG islands is generally associated with gene silencing, and with regard to our results, it is reasonable to suggest that EGR-1 cannot bind methylated GC-rich regions of promoters.

Here, we have performed the first study of *in vivo *occupancy changes of EGR-1 and its counterpart following stimulation. Our data show that both EGR-1 and SP1 binding occupancies change dramatically. EGR-1 binding may influence the occupancy of previous binding proteins, resulting in the reconstruction of the transcription factor complex and the induction of gene expression changes, although further experiments need to be performed in order to assess this. Of particular interest in this study was the reduction in occupancy of SP1 binding. A previous *in vitro *study reported that EGR-1 binding competed with SP1 binding because of similar consensus sequences [31]. Similar competition between the protein pair Hox and Smad have been reported [32]. We then speculate that EGR-1 could antagonize other GC-rich region binding proteins in addition to SP1. Since the most overrepresented sequence of EGR-1 binding regions is similar to that of not only SP1 but also SP3 (Figure 3b), SP3 may be a candidate competitor of EGR-1. SP3 has been reported to act as a dual-functional regulator whose activity is dependent on the context of DNA-binding sites in promoters. SP3 functions as a repressor when it is bound to a promoter through multiple DNA-binding sites, and as an activator when targeted to a promoter through a single DNA binding site [33]. Moreover, Leibermann and Hoffman reported that ectopic expression of EGR-1 abrogated the block in terminal differentiation impaired by Myc and E2F1, which can bind GC-rich consensus sequences [34,35]. We therefore guess that EGR-1 may influence the occupancy of Myc and E2F1 on their target gene promoters, as well as the down-regulation of Myc and E2F1 expression directly and/or indirectly.

The NGFI-A/EGR-1 binding proteins NAB1 and NAB2 have been reported as negative transcriptional cofactors capable of binding directly to EGR-1 and repressing EGR-1-mediated transcription [36,37]. In this study, enrichment of EGR-1 binding at 1 hour after PMA stimulation were observed in both *NAB1 *and *NAB2 *promoter regions (Figure 4). Moreover, the microarray data in FANTOM4 data sets showed that both *NAB1 *and *NAB2 *mRNA were induced until 2 hours after PMA treatment and decreased thereafter (Additional data file 7). These data strongly indicate that NAB1 and NAB2 are directly up-regulated by EGR-1 in THP-1 differentiation. Although NAB protein levels and the genome-wide locations of where EGR-1/NAB complexes bind have not been determined, our observation that *NAB *mRNAs are transiently expressed implies that direct repression by NAB proteins of EGR-1 transactivation during PMA stimulation may occur transiently. On the other hand, a current report showing that NAB2 interacts with the nucleosome remodeling and deacetylase complex suggests that a EGR-1/NAB complex could modify chromatin status [38]. Our investigation and further studies of epigenetic changes in THP-1 differentiation may contribute to elucidate the mechanisms of EGR-1/NAB transcriptional regulation.

Recently, a study of EGR-1 target genes in UV irradiated human prostate M12 cells was published [39]. To identify overlapping genes within both gene lists, we compared our 2,705 selected genes in PMA-stimulated THP-1 cells with 288 genes in UV irradiated M12 cells, and found 33 genes present in both lists. Interestingly, 19 of the 33 overlapping genes were closely related to nucleic acid binding, including transcription factor activity (*BLZF1*, *EGR2*, *ELF2*, *HLX1*, *ISL2*, *ZNF207*), transcription regulator activity (*CITED4*), DNA binding (*ORC6L*, *TAF6*, *ZNF345*, *ZNF565*), nucleic acid binding (*PINX1*), nucleotide binding (*GMPS*, *NME1*), histone (*H3F3A*), RNA splicing factor activity (*KHSRP*), RNA splicing (*PPIH*, *IVNS1ABP*) and RNA binding (*ADAR*). This enrichment strongly supports our conclusion that EGR-1 acts as an initiator of information transmission in cell events. Moreover, the observation that many genes do not overlap indicates that EGR-1 binding to DNA is dependent on cell type and/or stimulus. This observation also supports our notion that gene activation is important for EGR-1 binding.

Two independent lines of Egr-1 knockout mice have been reported [40,41]. Lee and colleagues [40] produced a deficient mouse line by homologous recombination using targeting vectors that localized at the beginning of the region encoding the first zinc finger in exon 2, whereas the mouse generated by Topiliko *et al*. [41] had the lacZ and neomycin genes inserted 50 bp upstream of the Egr-1 initiation codon in exon 1. Although both knockout mice were born normally, they exhibited individual abnormalities in growth, reproduction and long-term potentiation of neurons [42,43]. With regard to macrophage differentiation, a study that used the mice generated by Topiliko *et al*. argued that Egr-1 was a major positive modulator of macrophage differentiation [44], while Carter and Tourtellotte, who used the mice generated by Lee, showed that Egr-1 was neither essential for nor specific to monocyte/macrophage differentiation [45]. There are several possible explanations for these differences in phenotype between knockout mice lines. First, the location of the deletion in the gene may affect the expression of other genes, which is the case in prion gene (Prnp) knockout mice [46]. A couple of knockout mouse lines with targeted disruption of the coding gene *Prnp *were independently generated and two strikingly different phenotypes were reported. A group of knockout lines without prion protein expression produced ectopic Doppel, which is encoded by sequence 16 kb downstream of *Prnp *and has approximately 25% identity with the carboxy-terminal two-thirds of Prnp, and resulted in a cerebellar syndrome phenotype. Second, genes may be expressed from alternative start sites and avoid the impact of the targeting cassette insertion. In fact, the CAGE analysis from the FANTOM4 data has revealed that *EGR-1 *mRNAs are transcribed from a couple of alternative start sites (Additional data file 8). This result raises the possibility that alternative isoforms, which play a complementary or competitive role, may be produced from the *EGR-1 *locus.

## Conclusions

Here, we present the first genome-wide analysis of EGR-1 binding sites implicated in cell differentiation in human monoblastoma THP-1 cells. By combining genome context information, epigenetic profiling data and TSS identification, we conclude that EGR-1 mainly recognizes GC-rich consensus sequences of active genes in CpG islands. Using GOstat analysis, GO terms for EGR-1 target genes that were enriched included binding of nucleic acids and proteins. In addition, comparison with gene expression profiling data showed that immediately up-regulated genes are associated with EGR-1 binding in their promoter regions. These results confirm that EGR-1 acts as an initiator of information transmission in cell events. Moreover, we have demonstrated the first observation of *in vivo *occupancy changes of EGR-1 and SP1 following PMA stimulation. SP1 binding occupancies were dramatically changed near EGR-1 binding sites, suggesting that EGR-1 binding influences the occupancy of previous binding proteins. These observations may help explain why EGR-1 binding results in multiple responses to downstream genes.

## Materials and methods

### Cell culture and siRNA transfection

THP-1 cells were grown in RPMI1640 (Invitrogen, Carlsbad, CA, USA), 10% fetal bovine serum, 1% penicillin/streptomycin (Invitrogen), 10 mM HEPES (Invitrogen), 1 mM sodium pyruvate (Invitrogen) and 50 μM 2-mercaptoethanol (Invitrogen). THP-1 cells were incubated at 37°C in a humidified 5% CO_2 _incubator and differentiated with 30 ng/ml PMA (Sigma, St. Louis, MO, USA) up to 96 hours. Alexa Fluor 555 conjugated Stealth siRNA against EGR-1 (5'-UCUCCCAGGACAAUUGAAAUUUGCU-3') and a negative control siRNA were purchased from Invitrogen. For siRNA transfection, THP-1 cells were seeded in 6 cm dishes at a density of 1 × 10^6 ^cells/dish. Transfection was performed with 1.6 mg/ml (final concentration) of Lipofectamine 2000 (Invitrogen) and 20 nM (final concentration) of stealth siRNA by reverse transfection protocol in accordance with the manufacturer's instructions. Following siRNA treatment (48 hours), cells were incubated with PMA for differentiation. Cells were stained with Giemsa solution (Wako, Osaka, Japan) after fixing by methanol.

### Western blot analysis

Nuclear extracts taken at appropriate PMA stimulation times were prepared using NE-PER nuclear and cytoplasmic extraction reagents (Pierce, Rockford, IL, USA) according to the manufacturer's instructions. Total protein (20 μg) from each preparation were separated by SDS-PAGE in a 4-12% gradient NuPAGE polyacrylamide gel (Invitrogen) and transferred onto a PVDF membrane. Blots were incubated with rabbit anti-EGR1 polyclonal (#4152, Cell Signaling, Danvers, MA, USA) or rabbit anti-SP1 polyclonal (#07-645, Millipore, Billerica, MA, USA) antibodies and HRP-conjugated second antibodies and then were developed by the ECL Advance western blotting detection kit (GE Healthcare, Buckingamshire, UK). The chemiluminescence was recorded with a LAS-3000 luminescent image analyzer (Fujifilm, Tokyo, Japan).

### Chromatin immunoprecipitation assay

ChIP assays were performed as described previously [47] with minor modifications. The cells were cross-linked with 1% formaldehyde (Wako) for 10 minutes followed by addition of glycine (Wako) in phosphate-buffered saline at a final concentration of 125 mM. The cross-linked cells were collected by centrifugation and washed twice in cold 1× phosphate-buffered saline. The cells were sonicated for 5 minutes with a Branson 450 Sonicator to reduce the total DNA size from 150 to 600 bp (Additional data file 1c). The sheared chromatins were immunoprecipitated with anti-EGR1, anti-SP1, rabbit anti-acetyl-histone H3 Lys9 (#07-352, Millipore) antibodies or normal rabbit IgG (#12-370, Millipore) overnight at 4°C on a rotator. Immunoprecipitated samples were incubated with magnetic beads/Protein G (Invitrogen) for 1 hour at 4°C. Magnetic bead-antibody-chromatin complexes were washed once with low salt, high salt and LiCl buffers and twice with TE buffer. The chromatin complexes were eluted and incubated for 3.5 hours at 65°C to reverse the crosslink. To purify the DNA, RNA and proteins were digested with 20 μg/ml RNase and 100 μg/ml proteinase K, respectively. The DNA samples were recovered by phenol:chloroform:isoamyl alcohol extraction or QIAquick PCR purification kit (Qiagen, Valencia, CA, USA).

### LM-PCR, array hybridization and analysis of Affymetrix tiling array data

Immunoprecipitated DNA was blunted using 0.25 U/μl T4 DNA polymerase (Nippon Gene, Tokyo, Japan). Linker oligonucleotides (5'-accgcgcgtaatacgactcactataggg-3' and phosphate-5'-ccctatagtgagtcgtattaca-3') were annealed while the temperature was decreased gradually from 99°C to 15°C over 90 minutes. The blunted immunoprecipitated DNA sample was ligated with the annealed oligonucleotides by using 5 U/μl T4 DNA ligase (Nippon Gene). The cassette DNA fragments (60 μg/reaction) were amplified by using Blend *Taq *Plus (Toyobo, Osaka, Japan) with the linker-specific oligonucleotide 5'-accgcgcgtaatacgactcactataggg-3'. PCR amplification was done under the following conditions: denaturation at 95°C for 1 minute; 25 cycles of 95°C for 30 s, 55°C for 30 s, 72°C for 2 minutes; and a final extension at 72°C for 7 minutes. Amplified DNA was purified, fragmented with DNase I (Epicentre, Madison, WI, USA), and end-labeled with biotin-ddATP by using terminal deoxytransferase (Roche, Basel, Switzerland). Arrays were hybridized for 18 h at 45°C, washed, and scanned using the Affymetrix GeneChip System. The enriched and input samples were hybridized in triplicate. Raw array data were quantile normalized within three enriched and input technical replicates and scaled to a median feature intensity of 500. The genome coordinates of the 25-mer probes, originally based on version hg16 of the human genome, were converted to hg18. The positions of the probes on hg18 were determined by aligning the probe sequences to the human genome (hg18) using Vmatch [48]. For identification of high confidence EGR-1 binding sites on the human promoter arrays, we performed two independent experiments and chose clusters, where overlapping sites in biological replicates had over five consecutive array probes with a *P*-value < 1e-6.

### Real-time PCR for ChIP samples

For ChIP samples, real-time PCR was carried out using SYBR Premix ExTaq (Takara Bio Inc., Otsu, Japan) on the ABI PRISM 7500 Fast Real-Time PCR System (Applied Biosystems, Foster City, CA, USA) by denaturation at 95°C for 10 s, followed by running for 40 cycles at 95°C for 5 s and 62.5°C for 20 s. Occupancy values at each time point were calculated by determining the apparent immunoprecipitation efficiency (ratios of the amount of immunoprecipitated DNA over that of the input sample) and normalized to the level observed at a control region (Additional data file 9). Relative fold enrichment was calculated as the ratio fold enrichment of each sample to the 0 hour occupancy value. The primer sets used for real-time PCR analysis are shown in Additional data file 9.

### Data

The raw EGR-1 ChIP-chip data have been submitted to the Center for Information Biology Gene Expression database (CIBEX) with accession number [CIBEX:CBX71]. Illumina microarray gene expression data, Affymetrix whole tiling array data for H3K9ac enriched regions and promoter array data for SP1 binding regions are accessible through CIBEX accession numbers [CIBEX:CBX46], [CIBEX:CBX48], and [CIBEX:CBX43], respectively. All data, including that from deepCAGE, are also available via the Genome Network Platform [15]. The protein sequences of EGR-1 [Swiss-Prot:P18146], EGR-2 [Swiss-Prot:P11161], EGR-3 [Swiss-Prot:Q06889], EGR-4 [Swiss-Prot:Q05215] and WT-1 [Swiss-Prot:P19544] were used for motif analysis. In this paper, H3K9ac enriched loci are defined as a stretch of at least five consecutive array probes with a score (-log10 (*P*-value)) of over 30. High confidence SP1 enriched sites are defined as those with over five consecutive array probes in both biological replicates with a score ≥ 50.

## Abbreviations

CAGE: cap analysis gene expression; ChIP-chip: chromatin immunoprecipitation with genome tiling array; EGR: Early growth response gene; GO: Gene Ontology; H3K9ac: histone H3 lysine 9 acetylation; MEME: multiple Em for motif elicitation; PMA: phorbol 12-myristate 13-acetate; siRNA: small interfering RNA; TSS: transcriptional start site.

## Authors' contributions

AK designed and carried out experiments and wrote the paper. YT and MS carried out knockdown experiments. MT and EA carried out the *in silico *analysis of enriched sequence motifs and provided expertise in GO analysis. HM and TS supervised ChIP experiments and ChIP-chip analysis. HS and YH coordinated all efforts, supervised the project at all levels and consulted on project outcomes.

## Additional data files

The following additional data are available with the online version of this paper: a PowerPoint file containing three figures showing *EGR-1 *mRNA levels after siRNA mediated knockdown, differentiated THP-1 cells after siRNA mediated knockdown, and sonicated DNA (Additional data file 1); an Excel table listing myeloid related genes within predicted EGR-1 targets (Additional data file 2); a PowerPoint figure depicting the validation of EGR-1 enrichment by ChIP-real-time PCR analysis using EGR-1 antibody and normal IgG (Additional data file 3); PowerPoint Venn diagrams of the overlaps between EGR-1 binding sites, H3K9ac domains and CAGE tag clusters (Additional data file 4); a PowerPoint figure of SP1 protein levels in PMA-treated THP-1 cells (Additional data file 5); PowerPoint Venn diagrams of the overlaps between EGR-1 binding sites and SP1 binding sites (Additional data file 6); a PowerPoint figure showing *NAB1 *and *NAB2 *expression during THP-1 differentiation (Additional data file 7); a PowerPoint figure depicting TSSs in the *EGR-1 *gene locus (Additional data file 8); an Excel table listing real-time PCR primers for ChIP samples (Additional data file 9).

## Supplementary Material

Additional data file 1 **(a) **siRNA mediated knockdown of *EGR-1 *mRNA. *EGR-1 *mRNA were quantified using quantitative RT-PCR. *EGR-1 *mRNA levels were normalized to *GAPDH *mRNA and are presented relative to RNA levels in mock cells. RNA levels are representative of four independent experiments. **(b) **Effect of siRNA on *EGR-1 *in THP-1 differentiation. Phase contrast and fluorescence images were taken at the same time. Photographs show transfect efficiency indicated by Alexa Fluor 555 (upper) and typical morphological changes in *EGR-1 *or control siRNA transfected THP-1 cells at 48 hours after PMA stimulation (lower). The white arrows indicate differentiating THP-1 cells. **(c) **A sample of sonicated DNA. The sonication conditions were optimized to achieve enrichment of fragments between 150 and 600 bp in length. Click here for file

Additional data file 2 Myeloid related genes within predicted EGR-1 targets. Click here for file

Additional data file 3 PCR primers were designed for nine regions in selected clusters and six negative regions without enrichment in CpG islands. Data are relative fold enrichments, calculated by determining the apparent immunoprecipitation efficiency and normalized to the level observed at a control region (mean ± standard deviation, n = 2). Click here for file

Additional data file 4 Schematic Venn diagram representing the overlaps between EGR-1 binding sites, H3K9ac domains of each biological replicate and CAGE tag clusters. Click here for file

Additional data file 5 SP1 protein levels over a time course following PMA stimulation were observed by western blot analysis using a specific polyclonal antibody. Click here for file

Additional data file 6 Venn diagram showing the overlaps between EGR-1 binding sites and SP1 binding sites of each biological replicate. Click here for file

Additional data file 7 Quantile normalized *NAB1 *and *NAB2 *transcript levels were produced by Illumina Human Sentrix-6 bead chips v.2. Click here for file

Additional data file 8 deepCAGE tag clusters indicate transcriptional start sites in THP-1 differentiation. Click here for file

Additional data file 9 Real-time PCR primers for ChIP samples. Click here for file
