# Supplementary material for: Genome-wide investigation of in vivo EGR-1 binding sites in monocytic differentiation
Source: Genome Biol. 2009 Apr 19;10(4):R41. doi: 10.1186/gb-2009-10-4-r41 (PMC2688932; doi:10.1186/gb-2009-10-4-r41)
Supplement: Additional data file 1 — (a) siRNA mediated knockdown of EGR-1 mRNA. EGR-1 mRNA were quantified using quantitative RT-PCR. EGR-1 mRNA levels were normalized to GAPDH mRNA and are presented relative to RNA levels in mock cells. RNA levels are representative of four independent experiments. (b) Effect of siRNA on EGR-1 in THP-1 differentiation. Phase contrast and fluorescence images were taken at the same time. Photographs show transfect efficiency indicated by Alexa Fluor 555 (upper) and typical morphological changes in EGR-1 or control siRNA transfected THP-1 cells at 48 hours after PMA stimulation (lower). The white arrows indicate differentiating THP-1 cells. (c) A sample of sonicated DNA. The sonication conditions were optimized to achieve enrichment of fragments between 150 and 600 bp in length. [file gb-2009-10-4-r41-S1.ppt]

## Slide 1
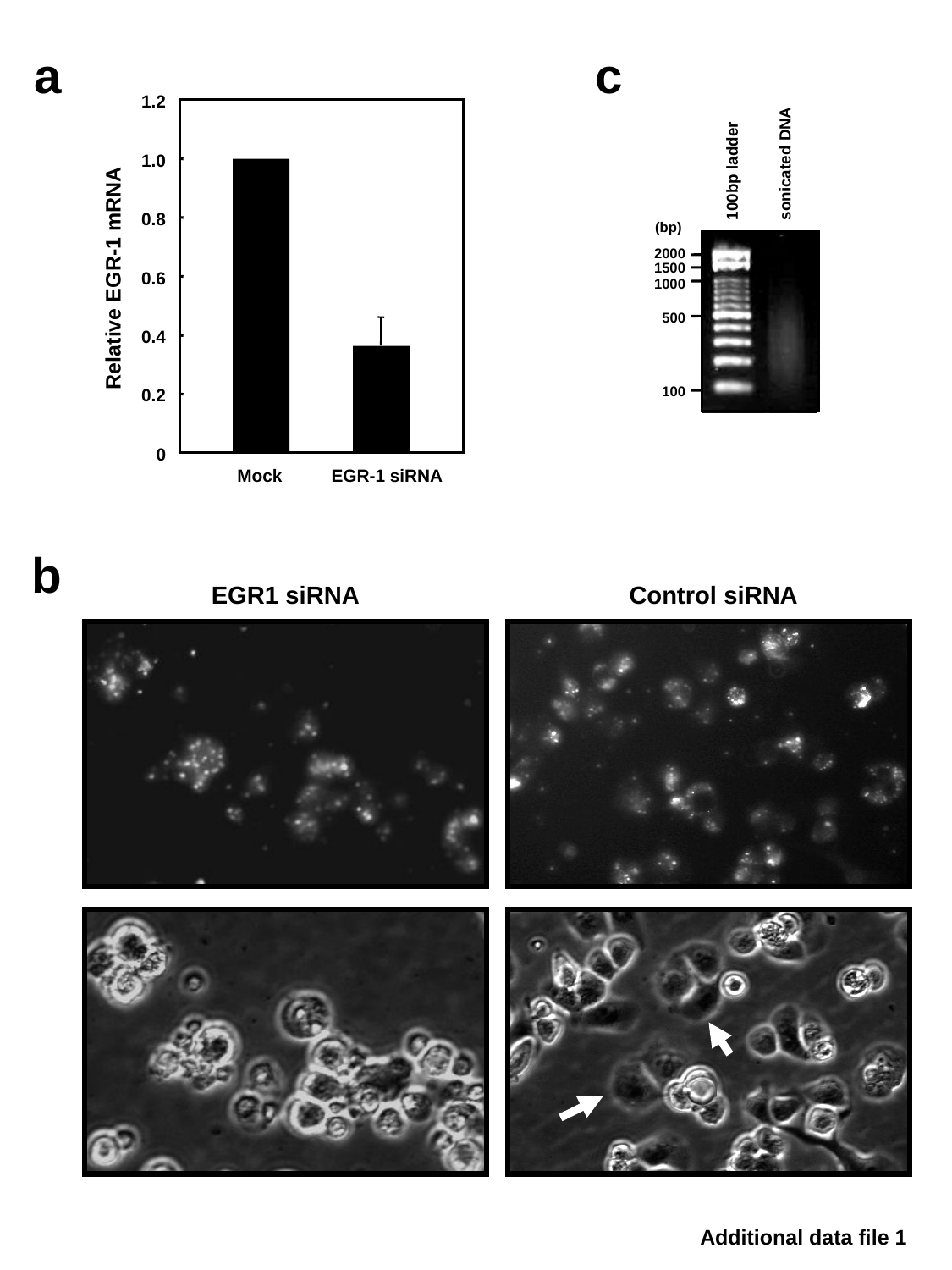

a
c
sonicated DNA
100bp ladder
(bp)
2000
1500
1000
500
100
1.2
1.0
0.8
Relative EGR-1 mRNA
0.6
0.4
0.2
0
Mock
EGR-1 siRNA
b
EGR1 siRNA
Control siRNA
Additional data file 1
