# Supplementary material for: Genome-wide investigation of in vivo EGR-1 binding sites in monocytic differentiation
Source: Genome Biol. 2009 Apr 19;10(4):R41. doi: 10.1186/gb-2009-10-4-r41 (PMC2688932; doi:10.1186/gb-2009-10-4-r41)
Supplement: Additional data file 3 — PCR primers were designed for nine regions in selected clusters and six negative regions without enrichment in CpG islands. Data are relative fold enrichments, calculated by determining the apparent immunoprecipitation efficiency and normalized to the level observed at a control region (mean ± standard deviation, n = 2). [file gb-2009-10-4-r41-S3.ppt]

## Slide 1
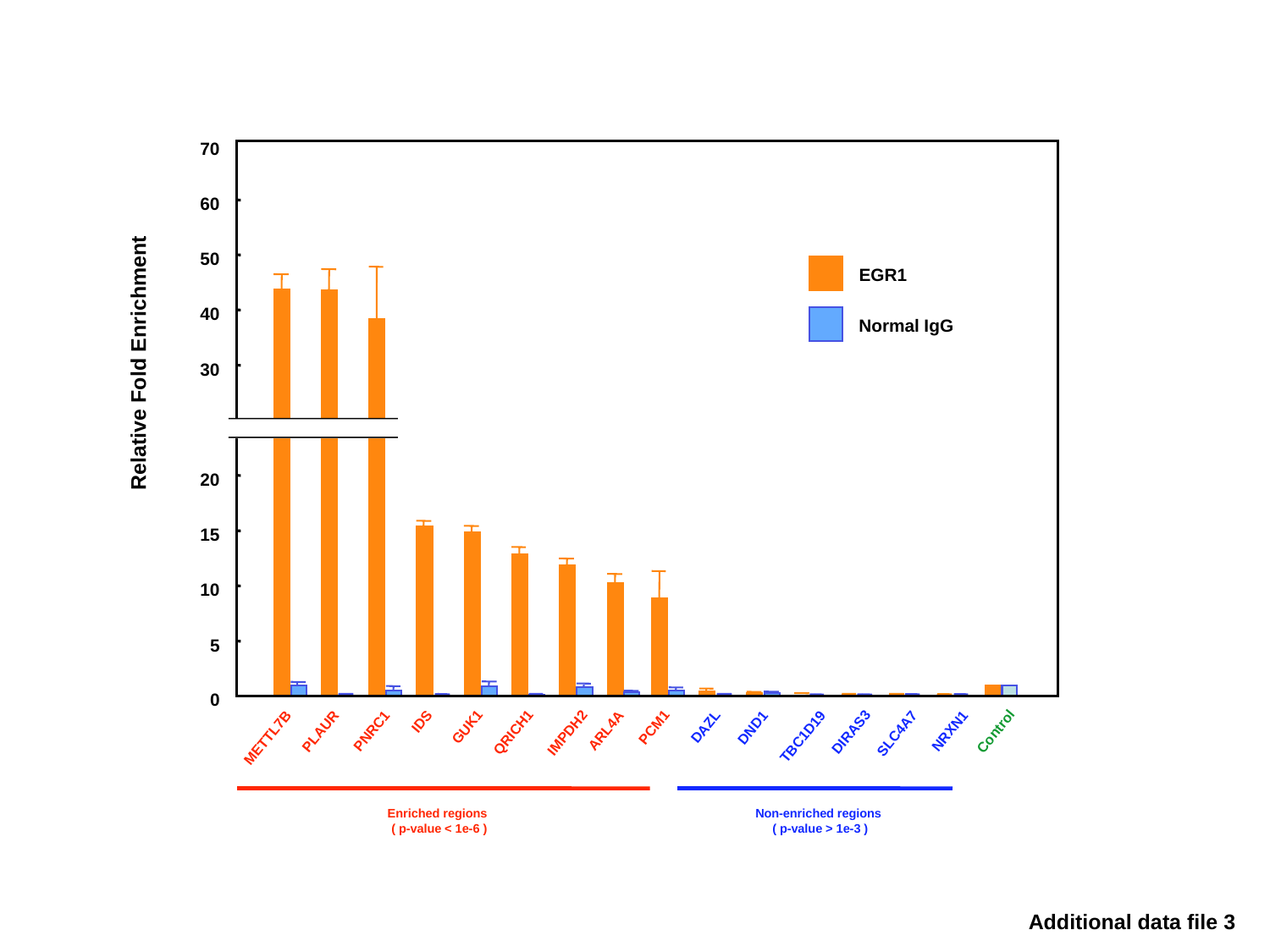

70
60
50
EGR1
40
Normal IgG
Relative Fold Enrichment
30
20
15
10
5
0
IDS
DAZL
GUK1
DND1
PCM1
NRXN1
PNRC1
ARL4A
Control
PLAUR
DIRAS3
QRICH1
IMPDH2
SLC4A7
TBC1D19
METTL7B
Enriched regions
( p-value < 1e-6 )
Non-enriched regions
( p-value > 1e-3 )
Additional data file 3
