# Supplementary figures and images for: Genome-wide investigation of in vivo EGR-1 binding sites in monocytic differentiation
Source: Genome Biol. 2009 Apr 19;10(4):R41. doi: 10.1186/gb-2009-10-4-r41 (PMC2688932; doi:10.1186/gb-2009-10-4-r41)

## Slide 1
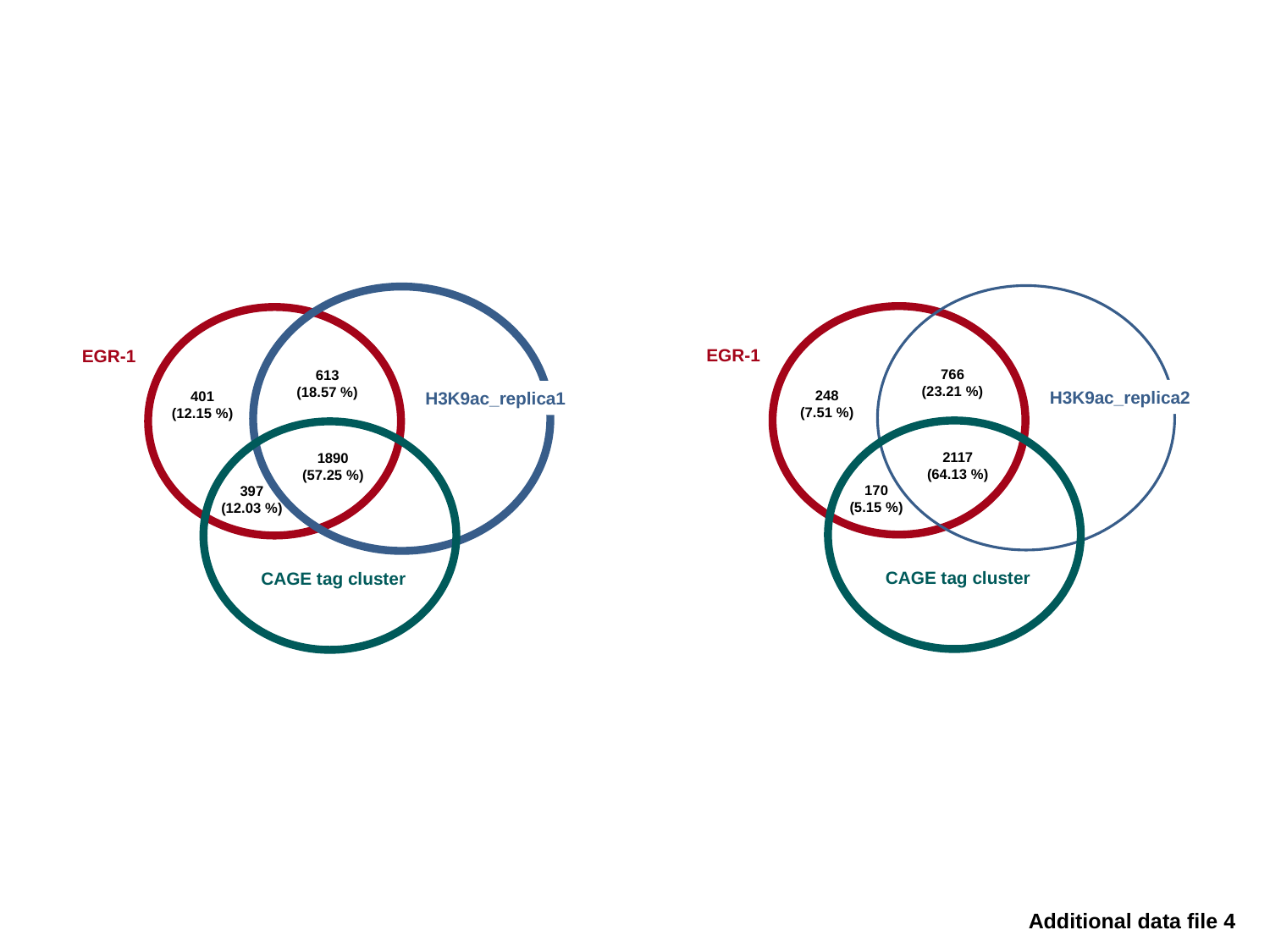

EGR-1
EGR-1
766
(23.21 %)
613
(18.57 %)
248
(7.51 %)
H3K9ac_replica2
401
(12.15 %)
H3K9ac_replica1
2117
(64.13 %)
1890
(57.25 %)
170
(5.15 %)
397
(12.03 %)
CAGE tag cluster
CAGE tag cluster
Additional data file 4

Supplement: Additional data file 4 — Schematic Venn diagram representing the overlaps between EGR-1 binding sites, H3K9ac domains of each biological replicate and CAGE tag clusters. [file gb-2009-10-4-r41-S4.ppt]

## Slide 1
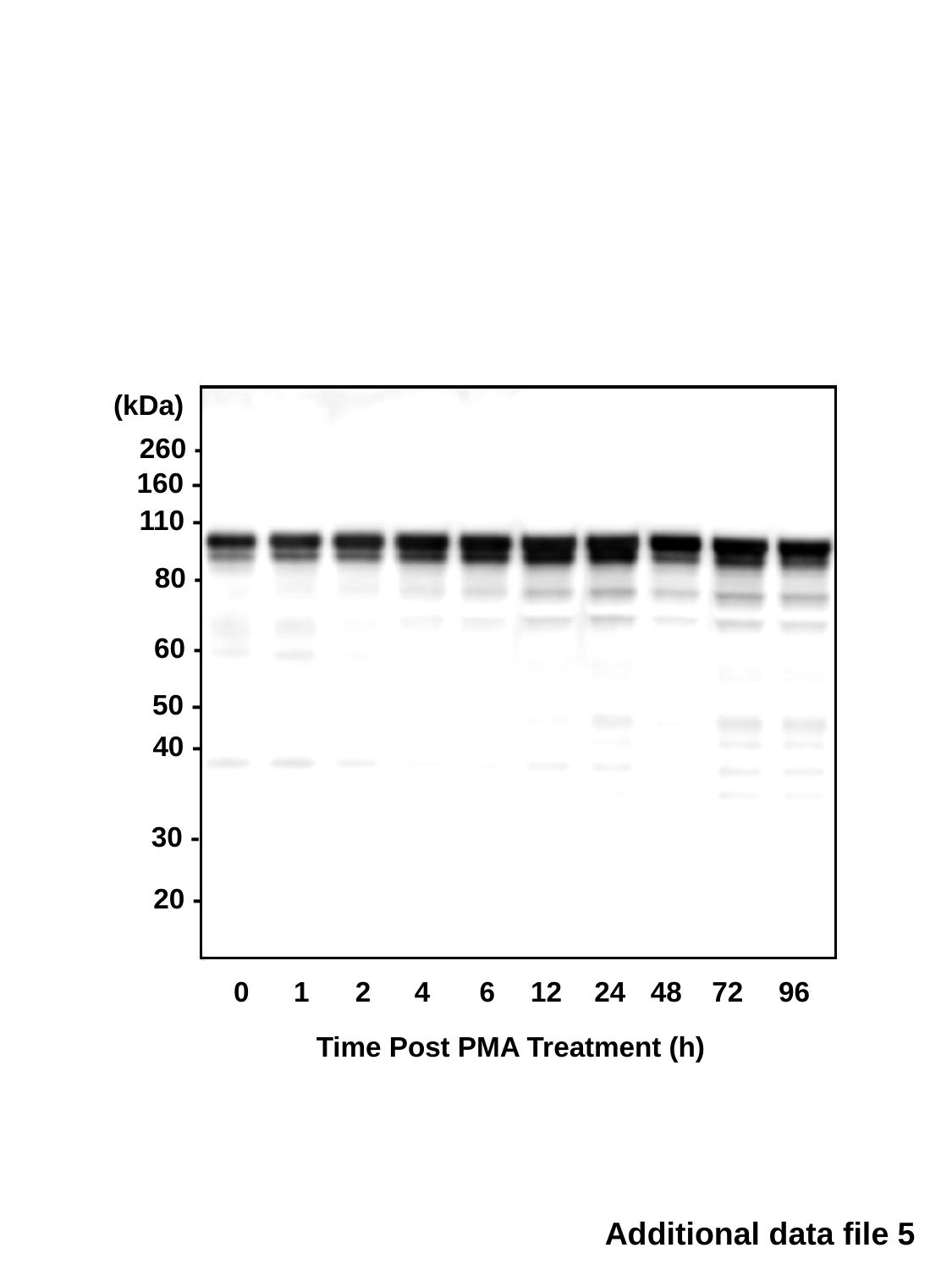

(kDa)
260 -
160 -
110 -
80 -
60 -
50 -
40 -
30 -
20 -
0
1
2
4
6
12
24
48
72
96
Time Post PMA Treatment (h)
Additional data file 5

Supplement: Additional data file 5 — SP1 protein levels over a time course following PMA stimulation were observed by western blot analysis using a specific polyclonal antibody. [file gb-2009-10-4-r41-S5.ppt]

## Slide 1
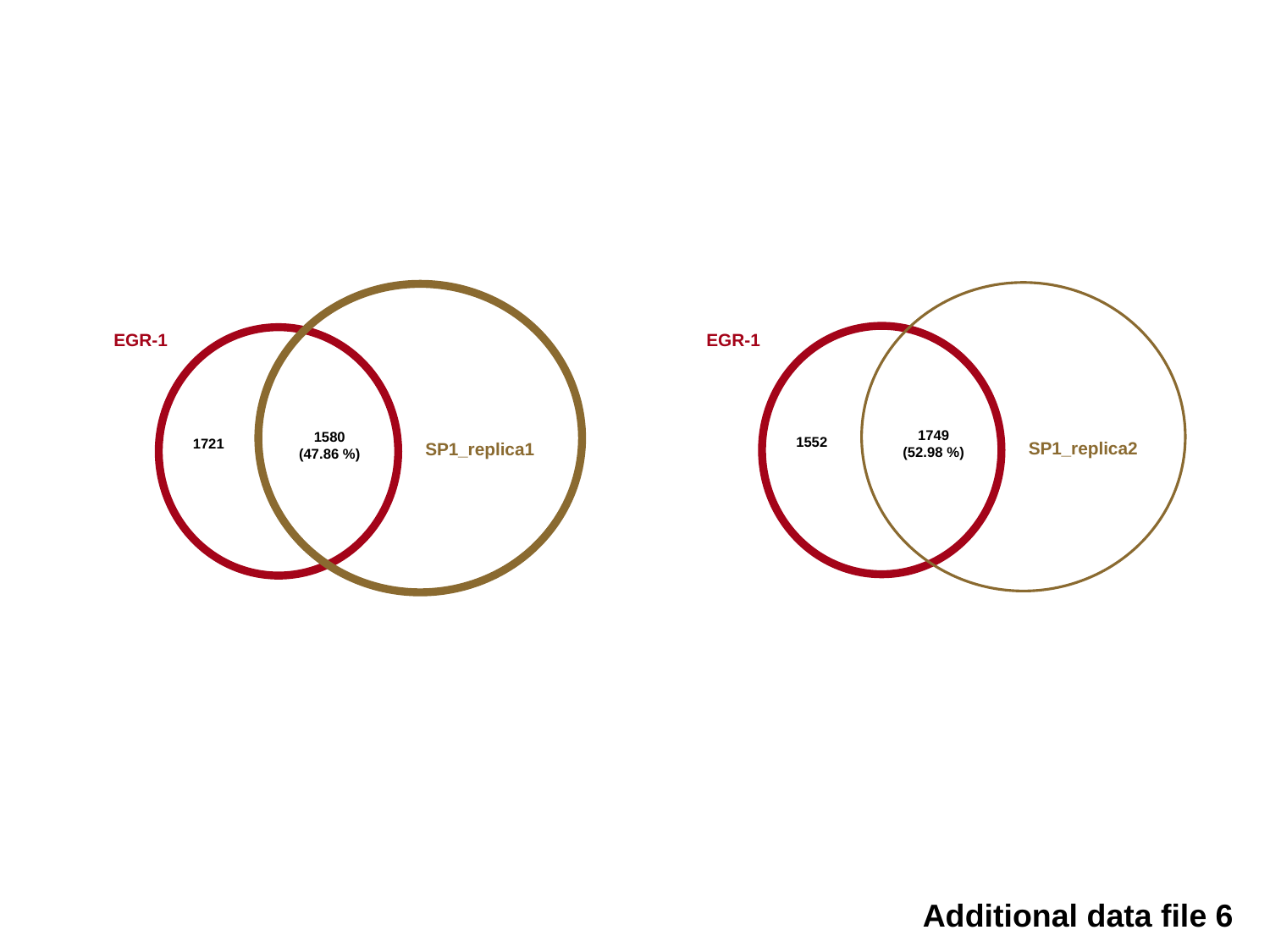

EGR-1
EGR-1
1749
(52.98 %)
1580
(47.86 %)
1552
1721
SP1_replica2
SP1_replica1
Additional data file 6

Supplement: Additional data file 6 — Venn diagram showing the overlaps between EGR-1 binding sites and SP1 binding sites of each biological replicate. [file gb-2009-10-4-r41-S6.ppt]

## Slide 1
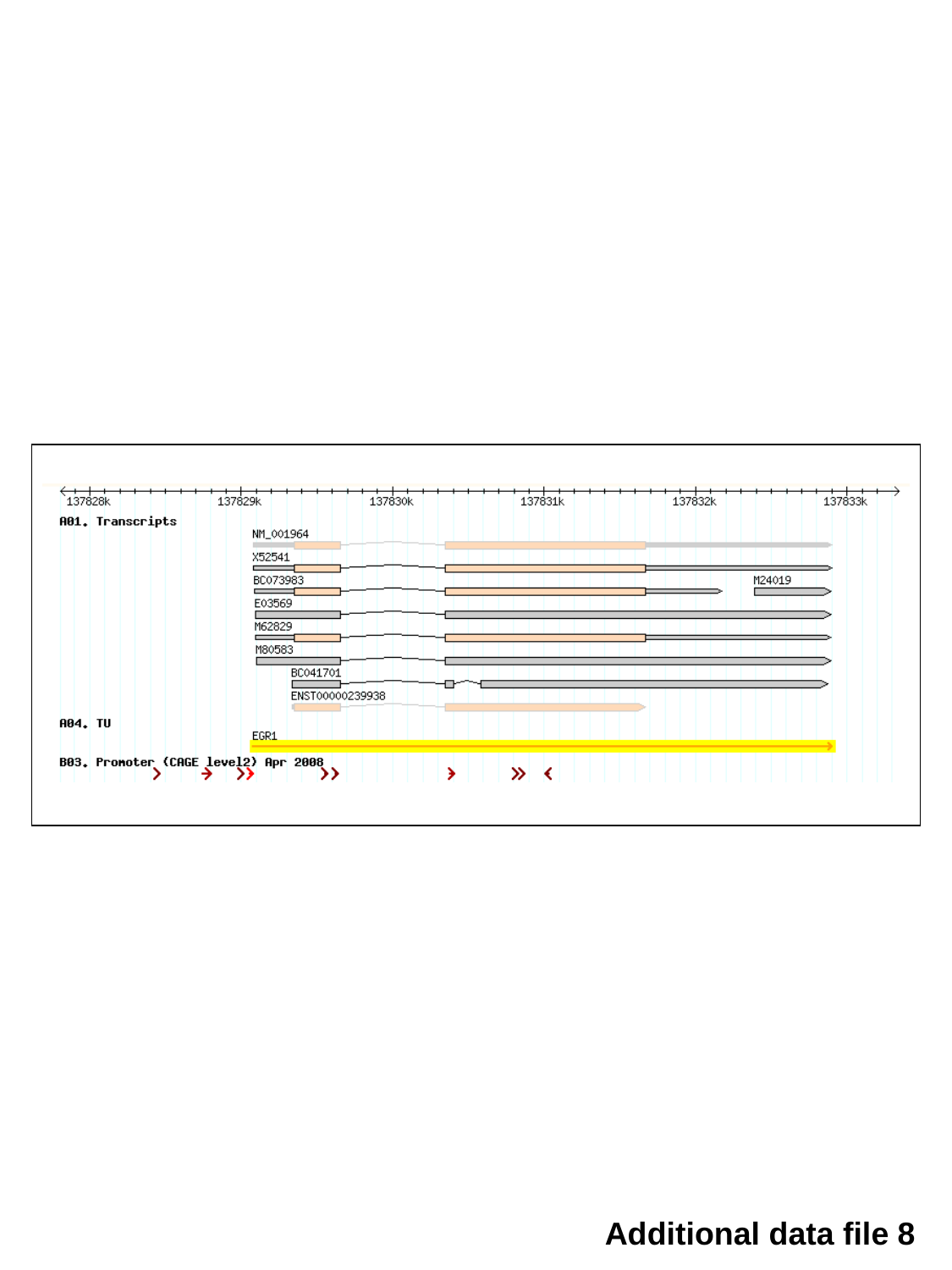

Additional data file 8

Supplement: Additional data file 8 — deepCAGE tag clusters indicate transcriptional start sites in THP-1 differentiation. [file gb-2009-10-4-r41-S8.ppt]
