# Supplementary material for: Genome-wide investigation of in vivo EGR-1 binding sites in monocytic differentiation
Source: Genome Biol. 2009 Apr 19;10(4):R41. doi: 10.1186/gb-2009-10-4-r41 (PMC2688932; doi:10.1186/gb-2009-10-4-r41)
Supplement: Additional data file 7 — Quantile normalized NAB1 and NAB2 transcript levels were produced by Illumina Human Sentrix-6 bead chips v.2. [file gb-2009-10-4-r41-S7.ppt]

## Slide 1
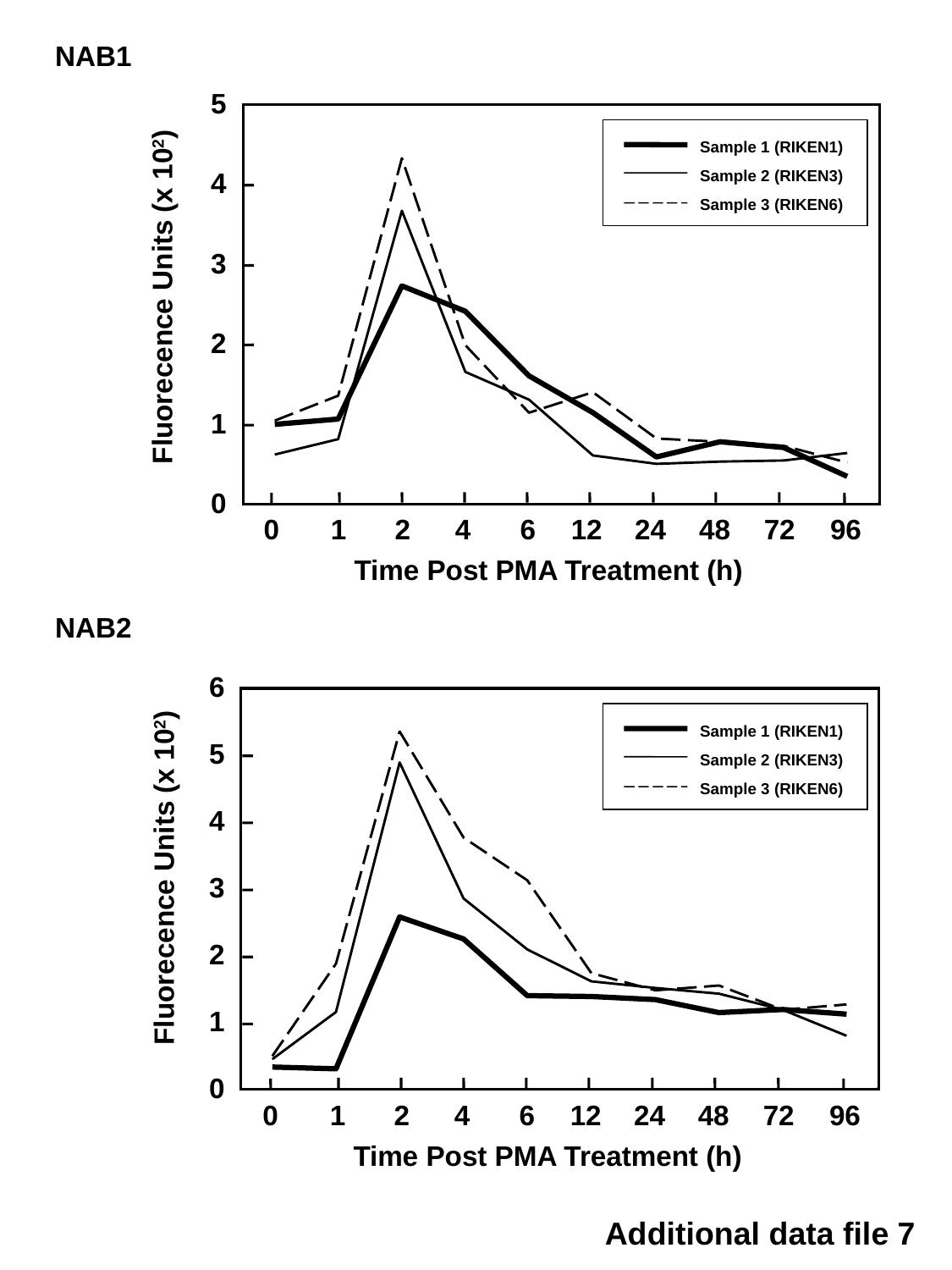

NAB1
5
Sample 1 (RIKEN1)
Sample 2 (RIKEN3)
Sample 3 (RIKEN6)
4
3
Fluorecence Units (x 102)
2
1
0
0
1
2
4
6
12
24
48
72
96
Time Post PMA Treatment (h)
NAB2
6
Sample 1 (RIKEN1)
Sample 2 (RIKEN3)
Sample 3 (RIKEN6)
5
4
Fluorecence Units (x 102)
3
2
1
0
0
1
2
4
6
12
24
48
72
96
Time Post PMA Treatment (h)
Additional data file 7
